# Racial Inequality in the Prime of Life: Infectious Disease Mortality in U.S. Cities, 1906–1933

**DOI:** 10.1017/ssh.2023.4

**Published:** 2023-06-13

**Authors:** Aja Antoine-Jones, James J. Feigenbaum, Lauren Hoehn-Velasco, Christopher Muller, Elizabeth Wrigley-Field

**Affiliations:** 1 University of California, Berkeley, Berkeley, CA 94720, USA,; 2 Boston University, Boston, MA 02215, USA,; 3 Georgia State University, Atlanta, GA 30303, USA,; 4 University of California, Berkeley, Berkeley, CA 94720, USA; 5 University of Minnesota, Twin Cities, Minneapolis, MN 55455, USA

**Keywords:** mortality, child mortality, racial inequality, epidemiological transition, population health, I15, J11, N32, O18

## Abstract

In the first half of the twentieth century, deaths from infectious disease, especially among the very young, fell dramatically in American cities. However, as infant mortality fell and life expectancy rose, racial inequality in urban infectious disease mortality grew. In this paper, we show that the fall in mortality and the rise in racial inequality in mortality reflected two countervailing processes. The dramatic decline in infant mortality from waterborne diseases drastically reduced the total urban infectious disease mortality rate of both Black and white Americans while having a comparatively small effect on the total racial disparity in urban infectious disease mortality. In contrast, the unequal fall in tuberculosis mortality, particularly in the prime of life, widened racial inequality in infectious disease mortality in US cities.

## Introduction

In the first half of the twentieth century, people living in the United States gained an average of 20 years of life. These life expectancy gains – concentrated among the very young – constituted the latter part of the United States’ *epidemiological transition* (Cutler 2004). However, as infant mortality declined and life expectancy rose, racial inequality in infectious disease mortality increased in American cities. The ratio of nonwhite-to-white deaths from infectious diseases in American cities rose from less than two-to-one at the beginning of the 20th century to nearly three-to-one in the early 1940s (Feigenbaum et al. 2022)^1^. In every year from 1906 to 1920, the infectious disease mortality rate of Black Americans living in cities exceeded that of urban white Americans during the 1918 influenza pandemic (Feigenbaum et al. 2019).

Were these two patterns related? One common explanation for racial inequality in mortality (Deaton 2013; Link and Phelan 1995) suggests that they were: white Americans may have gained access to medical innovations and public health infrastructure before Black Americans, leading racial inequality to rise as mortality rates fell unequally for Black and white Americans. Other research, in contrast, suggests that some public health innovations were equalizing (Troesken 2004, Anderson et al. 2021). Most famously, Troesken (2004) argues that because water systems were difficult to segregate, their introduction reduced racial inequality in death from waterborne diseases.

In this paper, we show that the fall in mortality and the rise in racial inequality in mortality in the first half of the twentieth century reflected two countervailing processes. The dramatic decline in infant mortality from waterborne diseases drastically reduced the total infectious disease mortality rate of both Black and white Americans living in cities and had a relatively small effect on the total racial disparity in urban infectious disease mortality. In contrast, the unequal fall in tuberculosis mortality, particularly in the age range of 10 through 30, widened racial inequality in mortality in US cities.

Our work follows the trail blazed by Preston and Haines (1991) in their classic book Fatal Years, celebrating its 30th anniversary in this issue. Using novel data from the Federal decennial censuses and vital statistics covering the Death Registration Area (DRA), Preston and Haines uncovered the alarmingly high rates of infant and child mortality that prevailed in early-20th-century America. Despite high levels of national wealth and literacy, nearly one of every five American children died before reaching five years old. Rather than a micro-sample of the census, we combine complete count census data from 1900 to 1940 with age-specific tabulations of the DRA vital statistics. We find that the story of the fatal years, featuring massive rates of infant and child mortality, ended at similar times and in similar ways for white and Black Americans. But racial inequality in urban infectious disease mortality persisted in the prime of life.

Our analysis proceeds as follows. We begin by reporting racial inequality in infectious disease mortality from 1906 to 1933 for eight age groups using data from all reporting cities. We find that racial inequality in the prime years of life was the largest and increased the most over time. Next, we show that while urban infant mortality from waterborne causes fell precipitously, racial inequality in these deaths remained comparatively low and changed relatively little over time. In contrast, racial inequality in urban deaths from tuberculosis was extremely high at all but the oldest ages, particularly among people younger than 30. The rate of death from tuberculosis among Black teenagers was so high that in many years it exceeded the rate of death from influenza among white teenagers during the 1918 influenza pandemic. Finally, we assess how much four broad causes of death contributed to racial inequality in mortality from infectious disease in US cities. At ages 10 to 30, deaths from tuberculosis accounted for roughly half of the nonwhite-white infectious disease mortality ratio.

## The epidemiological transition

Our period of analysis coincides with the late stages of the epidemiological transition in the United States. This transition was characterized by two broad changes in people’s life chances: a decline in deaths from infectious disease and a shift in the most common ages of death from young to old ages (Deaton 2013: 31).

The causes of this transition remain a subject of debate. One school of thought emphasizes the importance of improved nutrition (Fogel 2004; McKeown and Record 1962), especially the fact that increasing incomes enabled people to buy more and better food, which translated into better health. However, much of the early work on the transition was based less on precisely estimated effects of nutrition than on a failure to find strong evidence for other causes (Cutler et al. 2006).

Other scholars emphasize public health initiatives, particularly clean water and sewerage (Cutler and Miller 2005; Troesken 2004; Alsan and Goldin 2019) and local public health regulations driven by class struggle (Szreter 1988). Cutler and Miller (2005) found that water filtration drastically reduced typhoid, infant, and total mortality. Anderson et al. (2022) replicated this analysis after adding several cities, correcting transcription errors, and making other changes to its measurement and methods. They estimate nearly identical effects of filtration on typhoid mortality but revise the estimated effects on total mortality downward to the point of statistical insignificance. Precisely estimating the effects of public health interventions is challenging. Beach (2022: 7) notes that attempting to recover an average treatment effect of each clean-water intervention, as both Cutler and Miller (2005) and Anderson et al. (2022) do, can be misleading because “there was often one intervention that was important for improving water quality, but the intervention that mattered varied from city to city.” If a city began filtering its water before it began chlorinating it, for example, the estimated effect of chlorination could be muted because most of the mortality gains would have already taken place. Ultimately, despite disagreements about the magnitude of the urban mortality decline caused by clean-water technologies, all these authors agree about the importance of public health investments generally and clean-water technology specifically.

The epidemiological transition in the United States was concentrated in cities. In 1900, mortality rates were higher in American cities than in rural areas, but by mid-century, this gap had vanished (Haines 2001). Between 1900 and 1948, the share of urban deaths from infectious causes fell from a median of 37% to 6% (Feigenbaum et al. 2019).^2^

## Racial inequality in mortality in the early twentieth century

At the beginning of the 20th century, the Black child mortality rate far exceeded the white child mortality rate (Preston and Haines 1991: 96). This large total disparity, moreover, understated even larger disparities within urban and rural areas because most Black Americans lived in rural areas, where the risk of death from infectious disease was comparatively low. Over the next four decades, the evolution of racial inequality in infectious disease mortality was shaped by two countervailing trends: the disappearance of the urban mortality penalty and the large-scale migration of Black Americans to cities, where they were often pushed into segregated and crowded housing.

As discussed above, the introduction of clean-water technologies in US cities was an important contributor to the urban mortality decline. Because it was difficult to segregate urban water systems, these technologies also reduced racial inequality in mortality from waterborne diseases (Troesken 2004). Using data from 33 US cities from 1888 to 1920, Troesken (2004: 124) shows that water filtration had a large negative effect on the Black typhoid mortality rate, but a much smaller effect on the white typhoid mortality rate. Anderson et al. (2021) find similar effects of clean-water technologies on racial inequality in infant mortality. Filtration reduced both Black and white infant deaths, but chlorination reduced only Black infant mortality, thereby narrowing the Black-white infant mortality gap (Anderson et al. 2021). Although Black migrants began moving to cities when infectious death rates were higher in urban than rural areas, over time, this urban penalty weakened and ultimately reversed. Consequently, most of the gains in Black life expectancy between 1900 and 1940 were concentrated in cities (Troesken 2004: 4).

However, these gains in urban Black life expectancy between 1900 and 1940 were partially offset by the crowded, segregated, and poorly ventilated housing that many Black migrants were forced into. Such housing generated the ideal conditions for respiratory infections like tuberculosis to spread (Acevedo-Garcia 2000; Roberts 2009). Segregationists who used high rates of Black mortality to justify their cause (Stein 2012) thus got the causal relationship exactly backwards. Zelner et al. (2017) find that after the 1918 influenza pandemic, the estimated per-pulmonary-case risk of TB infection rose among Black Americans but not white Americans in 11 large US cities. These elevated transmission rates slowed the decline of tuberculosis mortality among Black Americans in cities, increasing racial inequality in death from tuberculosis. The nonwhite-to-white ratio in urban tuberculosis mortality jumped sharply from 2.5-to-1 during the influenza pandemic to more than 4-to-1 in the 1930s (Feigenbaum et al. 2022). In the early twentieth century, tuberculosis contributed more to the total racial disparity in infectious disease mortality in US cities than any other disease (Feigenbaum et al. 2022).

Unsegregated urban water systems and segregated, crowded housing should have pulled Black infectious disease mortality in different directions. On the one hand, clean-water technologies reduced racial inequality in typhoid and infant mortality. On the other, residential overcrowding likely increased racial disparity in death from tuberculosis. In the remainder of the paper, we use city-level data on deaths by cause and age to assess these predictions.

## Data

To study racial inequality in infectious disease mortality in the early twentieth century, we gather annual data on deaths by age and cause in all reporting US cities. These data come from published volumes of the *Vital Statistics of the United States*.^3^ We rely on the years 1906 to 1933 because these were the only years in the first half of the twentieth century when deaths were reported separately by age, cause, and racial classification. We combine counts of deaths by cause with age-and-racial-group-specific denominators from the IPUMS Restricted Complete Count Census Microdata (Ruggles et al. 2018) to construct cause-specific, age-specific mortality rates.

The *U.S. Vital Statistics* are the only data enabling us to study racial inequality in mortality by age and cause in the early 20th century, but these data have two notable limitations. First, they include only a small number of unrepresentative cities, listed in Table A.3. Because our primary interest is in comparing Black and white mortality within cities, our sample should accurately capture inequalities inside those particular cities, but may not generalize well to other cities, many of which had very small Black populations. Cities in the DRA reporting age and cause data by racial classification were selected twice over: they were included in the DRA and were further selected to report these specific data. Table A.4 compares the cities that ever appear in our sample to other cities based on their year of entry into the DRA. This comparison confirms that our sample contains substantially larger cities that had larger Black populations and were more likely to be in the South than other cities, either in the United States’ DRA or in the full urban population. The uneven distribution of the Black population across urban areas in this period means that we account for much of the urban Black population and those parts of the urban white population that shared cities with Black Americans, but far less of the urban white population as a whole. Feigenbaum et al. (2019) showed that, during most of the period we examine here, high mortality rates in southern cities were driven by extremely high mortality in those cities’ large Black populations. Until the mid-1920s, white mortality was similar across regions, but during the late 1920s and early 1930s, white infectious disease mortality was higher in southern cities than in other cities. Because our sample over-represents cities with high white mortality rates during the period when we document increasing racial inequality, it is likely that they understate racial inequality in urban infectious disease mortality at the national level.

Second, like all mortality series from this period, our data form an unbalanced panel. States were not required to report vital statistics until 1933, and cities entered the DRA in a staggered fashion over the period (Haines 2006), separately from and often well before their respective states. This was also true of DRA cities reporting deaths by age and racial classification (as shown in Table A.3). Because the results reported below document changes in racial inequality during the 1920s, it is reassuring that no cities were added during that decade.^4^ Therefore, the changes we document do not simply reflect changes in which cities are included in the sample.

Throughout the paper, we report the median infectious disease mortality rate. In the appendix, we reproduce our results using the median all-cause mortality rate. When calculating the median mortality rate, we weight by the city population to capture the mortality risk experienced by the median person. In the appendix, we show that our results are substantively identical if we use weighted means instead.

We present median mortality rates by age using eight age groups: infants (under 1), ages 1–4, ages 5–19, ages 10–19, ages 20–29, ages in the 30s and 40s, ages in the 50s and 60s, and ages 65/70 and up. The Vital Statistics made a slight change in the age groupings it reported between 1921 and 1922. While age groupings under 29 were unaffected, age groups over 30 do change. For example, whereas we see an aggregation of deaths among 30 to 49-year-olds before 1922, that group changes to 30 to 44-year-olds after 1922. Table A.1 shows the specific ages included in each age group for the periods 1906–1921 and 1922–1933. We construct denominators to match the age groupings used in each year. In all figures involving age groups 30 and over, we indicate the year of the age-grouping change with a vertical gray line.

In addition to reporting infectious disease mortality rates, we also report rates of death from specific causes. Table A.2 lists the causes included in each major grouping we consider – respiratory (pneumonia, influenza, and bronchitis), tuberculosis (all forms), waterborne/foodborne, and childhood illnesses. We study these causes for several reasons. First, diarrheal deaths from waterborne and foodborne diseases were the largest killer of children during this period (Preston and Haines 1991). As discussed above, clean-water technologies also caused infant and childhood deaths to decline (Alsan and Goldin 2019; Beach et al. 2016; Ferrie and Troesken 2008), and these declines affected both Black and white children (Anderson et al. 2021; Troesken 2004). Respiratory causes (influenza, pneumonia, and bronchitis) and tuberculosis, in contrast, should have affected Black and white Americans differently due to their differential exposure to segregated and crowded housing. Finally, we consider the major childhood causes of death (diphtheria, measles, whooping cough, scarlet fever, and smallpox) reported in Preston and Haines 1991 to assess the extent to which these contributed to the total racial disparity in infectious disease mortality.

## Results

**Figure 1.**
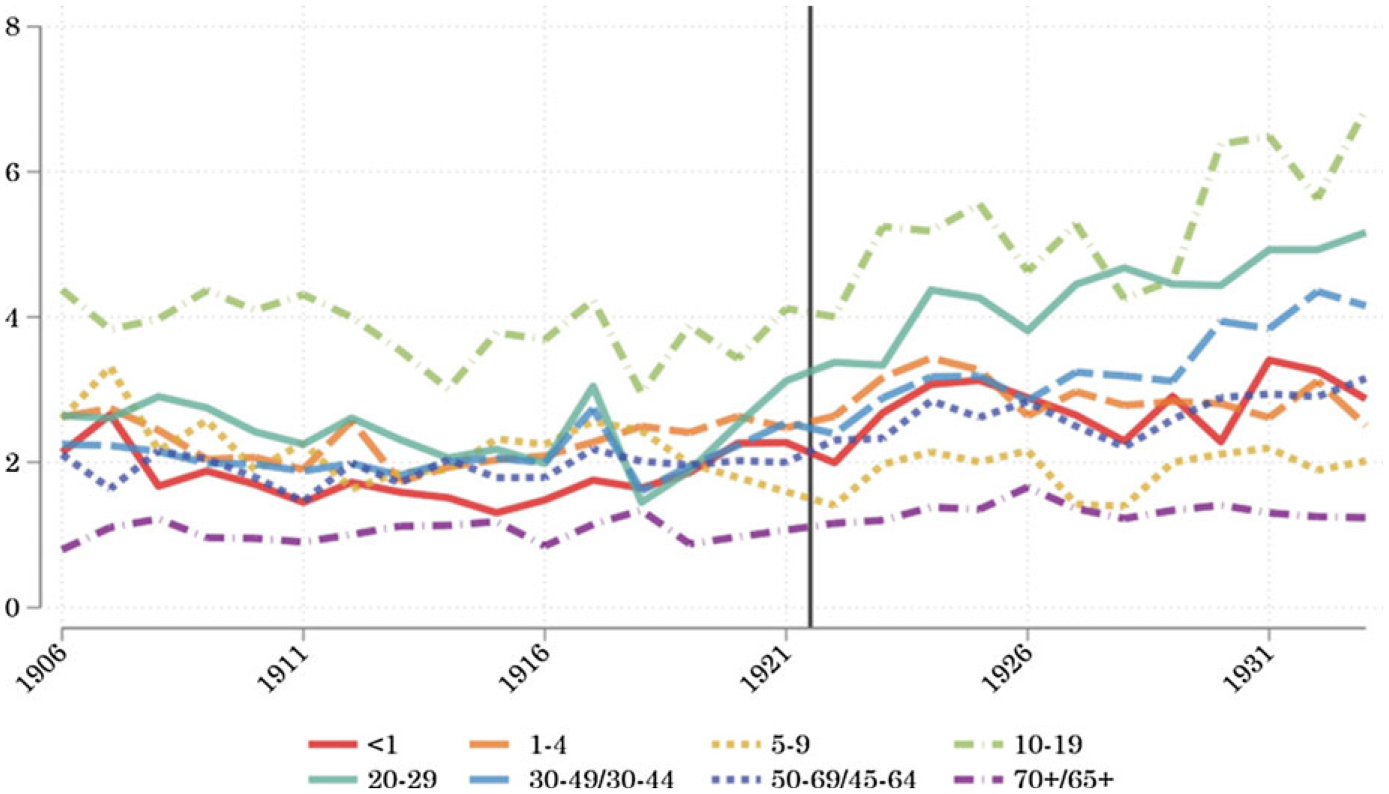
Age-specific *infectious* disease mortality disparity. *Notes:* Ratio of nonwhite-to-white mortality from infectious diseases in U.S. cities, 1906–1933. The vertical line shows the change in age classifications from 1921 to 1922, which only applies to ages 30 and up. The line only appears on graphs where the age-grouping changes after 1921. *Sources:* Mortality data by age and racial classification from published volumes of the *Vital Statistics of the United States*. Racial-group- and age-specific population counts (for the denominators) from the IPUMS Restricted Complete Count Census data.

Figure 1 reports the ratio of nonwhite-to-white urban mortality from infectious diseases from 1906 to 1933 among eight age groups.^5^ Throughout the results, we refer to this ratio as racial inequality in mortality. The figure shows that in all but the very oldest age groups, nonwhite Americans’ rate of death from infectious disease was much higher than that of white Americans.

However, racial inequality in mortality varied widely across age groups. It was largest in the teen years through midlife, when white mortality was extremely low. At ages 10–19, the nonwhite-to-white ratio lept from 3:1 in 1918 to nearly 7:1 in 1933. A similarly pronounced increase in the ratio of nonwhite-to-white mortality took place among people in their 20s, 30s, and 40s.

**Figure 2.**
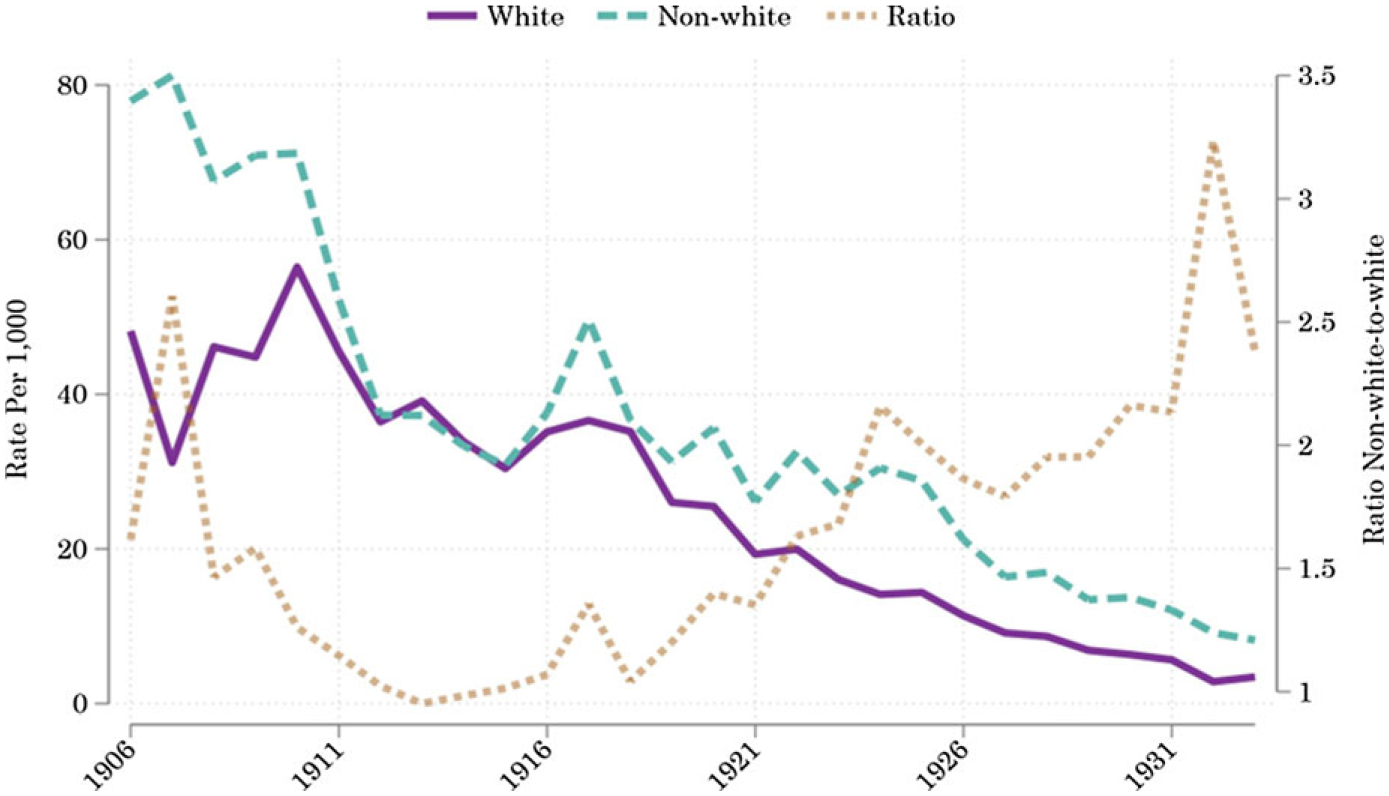
Infant mortality due to waterborne causes of death. *Notes:* Weighted medians of infectious mortality in U.S. cities over 1906–1933. Mortality rates per 1,000 age-racial-group-specific population. *Sources:* Mortality data by age and racial classification from published volumes of the *Vital Statistics of the United States*. Racial-group- and age-specific population counts (for the denominators) from the IPUMS Restricted Complete Count Census data.

Which causes of death were responsible for these wide inequalities? Figure 2 clearly demonstrates the importance of waterborne causes to the infant mortality decline. The nonwhite infant mortality rate from waterborne causes fell from 77.9 to 8.2 per 1,000 persons between 1906 and 1933. The white infant mortality rate fell from 48.2 to 3.5 per 1,000 persons over the same years. Racial inequality in infant mortality from these causes hovered between roughly 1 and 3 over the period. Figure 2 thus confirms that both massive declines in infant mortality and comparatively low levels of racial inequality in infant mortality were achievable, even in the early 20th century. The relative stability in the nonwhite-to-white ratio, as compared to the ranges of ratios in Figure 1, is likely attributable to the fact that urban water systems were less segregated than urban housing.

**Figure 3.**
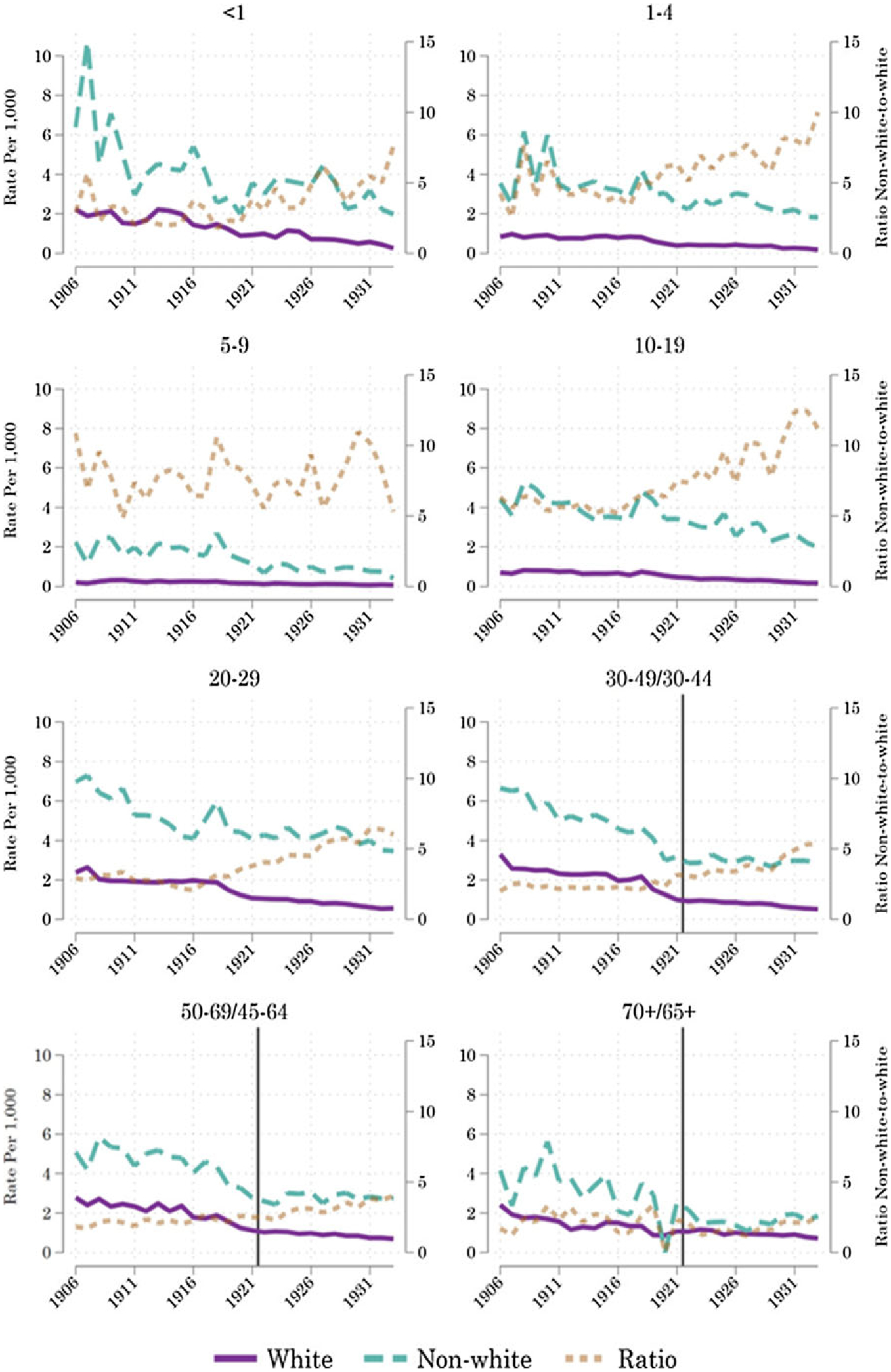
Tuberculosis age-specific racial disparity. *Notes:* Weighted medians of infectious mortality in U.S. cities over 1906–1933. Mortality rates per 1,000 age-racial-group-specific population. The vertical line shows the change in age classifications from 1921 to 1922, which only applies to ages 30 and up. The line only appears on graphs where the age-grouping changes after 1921. *Sources:* Mortality data by age and racial classification from published volumes of the *Vital Statistics of the United States*. Racial-group- and age-specific population counts (for the denominators) from the IPUMS Restricted Complete Count Census data.

In contrast, Figure 3 shows that racial inequality in the risk of death from tuberculosis was vast, rising to ratios in the range of 7-to-one to above 10-to-one in the 1930s for some age groups. The tuberculosis death rate among both nonwhite and white infants was much lower than the death rate from waterborne causes. As we move up the age range, however, racial inequality in the tuberculosis death rate rises. Thus, the shared mortality gains in infancy from waterborne causes shown in Figure 2 were partially offset by rising racial inequality in tuberculosis mortality in the prime of life.

**Figure 4.**
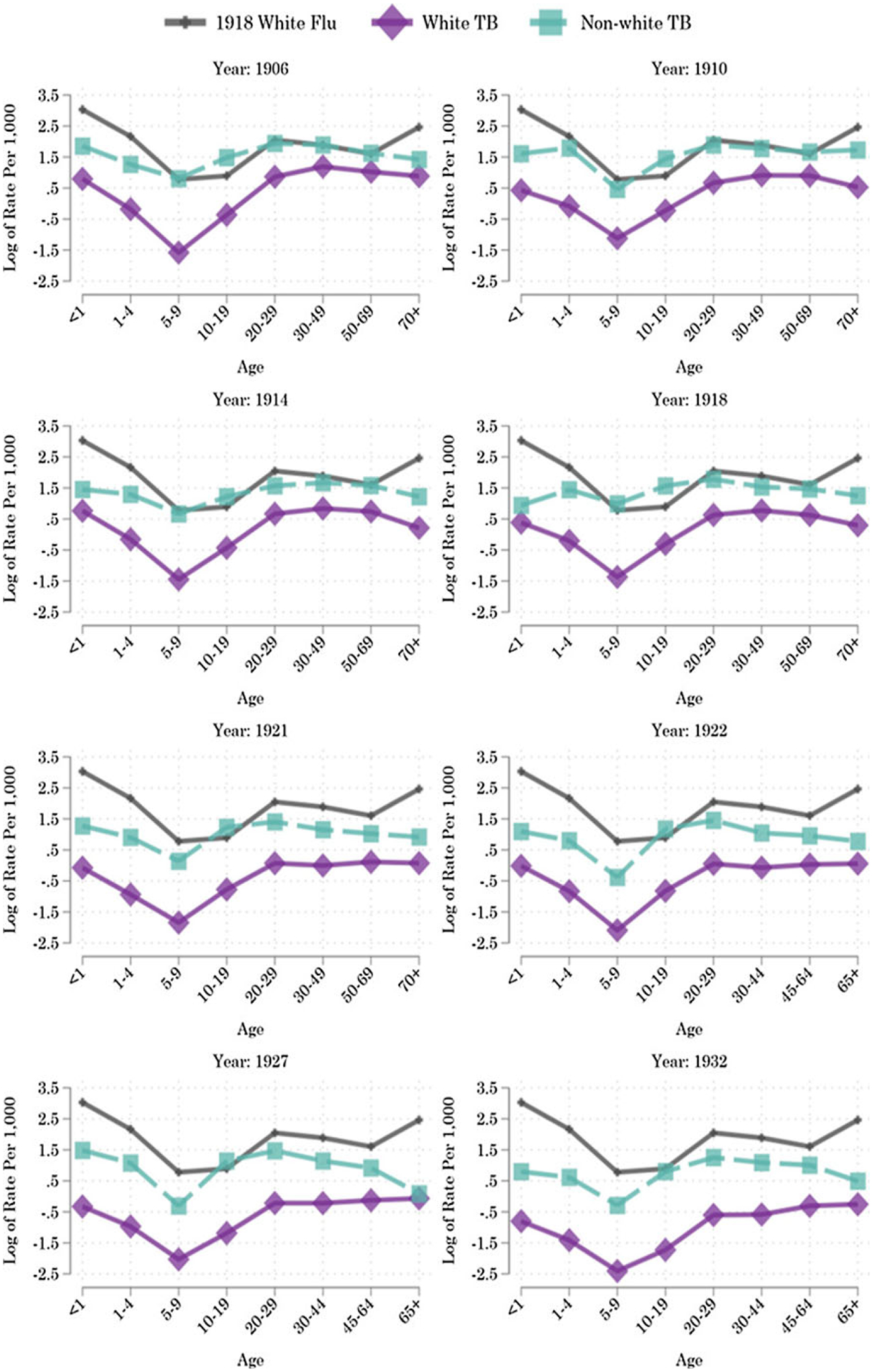
Tuberculosis mortality by racial classification and age. *Notes:* Weighted medians of infectious mortality in U.S. cities over 1906–1933. Mortality rates per 1,000 age-racial-group-specific population. *Sources:* Mortality data by age and racial classification from published volumes of the *Vital Statistics of the United States*. Racial-group- and age-specific population counts (for the denominators) from the IPUMS Restricted Complete Count Census data.

Feigenbaum et al. (2022, 2019) compared the typical level of urban Black infectious disease mortality in the early twentieth century to the white level of urban infectious disease mortality during the influenza pandemic. Here we show that the analogy extends to Black Americans’ extreme risk of dying from tuberculosis in the prime of their lives. Beyond its sheer scale, one feature of the 1918 influenza pandemic that made it historically unprecedented was its unusual age-mortality pattern. In addition to the very young and very old people who were most likely to die of influenza in non-pandemic years, the pandemic struck down many young adults (Beach et al. 2022). Figure 4 shows that in many years in the early twentieth century, Black teenagers’ risk of death from tuberculosis alone was so high that it exceeded white Americans’ risk of death from influenza during the 1918 influenza pandemic.

Table 1 shows how much deaths from respiratory, tuberculosis, waterborne, and childhood illnesses contributed to racial inequality in infectious disease mortality at different ages. For each age group, we set Black Americans’ rate of death from a given cause to the corresponding white rate of death from that cause, then recalculate racial inequality in mortality from all infectious diseases. The first column shows that deaths from tuberculosis were responsible for roughly half of the total racial disparity in deaths from infectious disease at ages 10 to 29. No single cause contributed more to age-specific racial inequality in death from infectious disease than tuberculosis.

## Implications

The numbers we report here are inadequate to the scale and depth of suffering they imply. Beach et al. (2022: 48) note that death among young adults and adults in midlife during the 1918 influenza pandemic “was particularly notable and tragic for individuals losing parents, spouses, and breadwinners.” After the pandemic, infectious disease deaths in the prime of life became much less common for white Americans. Although the absolute decline in tuberculosis mortality over time meant that the suffering of bereavement became rarer, Black Americans continued to “die in great numbers without a crisis ever being declared” (Hartman 2020).

With a persistent risk of contracting tuberculosis, Black people wrote to doctors and health advice columnists asking whether they should delay marrying and having children. In 1932, a young man who had contracted tuberculosis asked an advice columnist in the *Pittsburgh Courier* whether he should marry his partner: “Do you think I ought to tell her that I cannot marry her? You see, we would have puny children and I would not live long enough to educate them” (Strong 1932). Responding to a similar question in the *Chicago Defender*, Dr. A. Wilberforce Williams argued that “Marriage and tuberculosis are not good partners.” He advised the inquirer to postpone his marriage “for one year at least, after you have been cured of all sign and symptoms of tuberculosis” (Williams 1916). An obituary for William “Social” Morris published in the *Defender* in the same year noted that he succumbed to tuberculosis just five months after getting married (“Social” Morris Dead 1916).

Tuberculosis robbed many Black children of parents and economic security. “Tuberculosis is guilty of many a mother’s early death,” noted Elvin Elliott Rawlins, a Black physician who wrote a weekly medical column in the *New York Amsterdam News* (Rawlins 1923). “A high death rate in the middle of life,” was also “a serious economic problem for the race,” argued R. Maurice Moss of the Urban League (Moss 1926). Even in the absence of deaths from tuberculosis, the fear of contagion may have led some Black workers to lose their jobs. In 1928, a white citizen of Kansas told a newspaper that “Immediately after the newspapers came out with their statement of health conditions among Negroes, as shown by the tuberculosis survey of Dr. W. J. Thompkins, every one of my neighbors discharged every Negro servant in his employ” (False Survey Made Women Lose Jobs 1928). The economic consequences of death and infection in the prime of life thus may have been an important contributor to the Black-white gap in family income (Jácome et al. 2021).

## Conclusion

In American cities in the early twentieth century, Black and white Americans had far more equal access to safe water than to safe housing (Roberts 2009; Troesken 2004). This fact helps to explain several features of the evolution of racial inequality in infectious disease mortality from 1906 to 1933. The provision of clean-water technologies was a major success story not just for average life expectancy, but also for improving racial equality in mortality. Deaths from waterborne causes fell dramatically among both nonwhite and white Americans, particularly the very young, without drastically increasing the nonwhite-white ratio in infectious disease mortality. This result adds further weight to several previous studies showing how powerful the provision of universal public health infrastructure can be (Alsan and Goldin 2019; Anderson et al. 2021; Anderson et al. 2022; Beach et al. 2016; Beach 2022; Cutler and Miller 2005; Ferrie and Troesken 2008; Troesken 2004).

The lack of universal access to safe housing, in contrast, could help explain the rise in racial inequality in infectious disease mortality during the late stages of the United States’ epidemiological transition. As rural Black Americans moved to cities, they were routed through violence and policy into poorly ventilated and crowded housing. The estimated tuberculosis transmission rate rose among urban Black Americans at the same time as it fell among urban white Americans, leading to rising racial inequality in tuberculosis mortality (Zelner et al. 2017). Tuberculosis deaths among Black Americans were concentrated in the prime of life (Roberts 2009: 24–26). In many years, Black teenagers faced a pandemic-level risk of dying from tuberculosis alone.

The persistence of Black deaths in the prime of life was a tragedy in its own right, but it also had consequences for many other dimensions of Black Americans’ lives. The risk of dying from tuberculosis may have influenced some Black Americans’ decisions about whether to start families. More Black than white adults would have experienced widowhood and more Black than white children would have experienced the death of a parent because of the ongoing threat of death from tuberculosis. Black family incomes would have been cut more than white family incomes because of parental deaths. And the threat of young adult death that made the 1918 influenza pandemic so terrifying persisted for Black Americans at least through the early 1930s (Beach et al. 2022). With COVID-19 still spreading around us, we have all gained a tragic glimpse into what it is like to live in a pandemic. It will take the work of future historians to document what it was like to live with a pandemic pattern of mortality in every year.

## Supplementary Material

Supp material

## Figures and Tables

**Table 1.** Contribution to age-specific racial disparity (%)

*Average Across Years*				
*Age Category*	Tuberculosis	Waterborne	Pneumonia & Flu	Childhood
Infant	2.8	11.1	30.2	6.1
1–4	13.1	5.2	30.9	3.6
5–9	31.8	4.1	13.1	−2.6
10–19	55.5	2.8	11.9	−0.2
20–29	44.5	1.2	13.6	−0.1
30–49/30–44	29.3	0.8	16.4	−0.0
50–69/45–64	14.4	0.8	17.1	−0.0
65+	4.8	−1.3	1.0	−0.1
All	24.6	3.0	18.1	1.4

*Notes*: We calculate the adjusted disparity by setting the nonwhite age-specific mortality rate to the white age-specific mortality rate for each cause, and then recalculating the age-specific infectious disease mortality rate with that adjusted mortality rate. Then the percent of the racial disparity explained by each cause (for each age) is calculated by comparing the observed disparity to the adjusted disparity: $100*\frac{\text{Observered Disparity}-\text{Adjusted Disparity}}{\text{Observered Disparity}}$ where the disparity is the ratio of nonwhite-to-white infectious disease mortality for each age. Specific values calculated by taking median across city and average across years.

## Footnotes

1 Our mortality data enable us to measure deaths among people classified as white and nonwhite. Black Americans made up the vast majority of people classified as nonwhite during our period (Gibson and Jung 2002).

2 Deaths from infectious disease generally constituted a larger portion of total mortality in the Black population, even as that portion fell for both Black and white Americans. The median city’s share of all deaths that were from infectious disease fell from more than 50% in the early 1900s to around 40% in the 1920s and 1930s for Black Americans, compared with around 40% in the early 1900s to around 25% by the 1930s for white Americans.

3 The *Vital Statistics* began aggregating data collected by a limited number of states and cities in 1900 (Haines 2006). All cities in the United States were included by 1933. See Hacker et al (this issue) and Steckel (1988) for innovative attempts to measure mortality in the 19th century via linking across censuses, before the establishment of the permanent Census Office (later the Census Bureau) in 1902 and before the general adoption by the Census of the International Classification of Causes of Death.

4 Five cities were added in 1920, and seven were added in 1930. No cities were added in between those years.

5 The vertical line shows the change in age classifications from 1921 to 1922, which only applies to ages 30 and up. The line only appears on graphs where the age-grouping changes after 1921.

## References

[R1] Acevedo-Garcia Dolores (2000) “Residential segregation and the epidemiology of infectious diseases.”Social Science & Medicine 51 (8): 1143–61. 11037206 10.1016/s0277-9536(00)00016-2

[R2] Alsan Marcella, and Goldin Claudia (2019) “Watersheds in child mortality: The role of effective water and sewerage infrastructure, 1880–1920.” Journal of Political Economy 127 (2): 586–638. 31073249 10.1086/700766 PMC6502471

[R3] Anderson D. Mark, Charles Kerwin Kofi, Rees Daniel I., and Wang Tianyi (2021) “Water purification efforts and the black-white infant mortality gap, 1906–1938.” Journal of Urban Economics 122: 103329.

[R4] Anderson D. Mark, Charles Kerwin Kofi, and Rees Daniel I. (2022) “Reexamining the contribution of public health efforts to the decline in urban mortality.” American Economic Journal: Applied Economics 14 (2): 126–57.

[R5] Anonymous (1916) “‘Social’ Morris Dead.” Chicago Defender, December 2.

[R6] Anonymous (1928) “False Survey Made Women Lose Jobs.” Afro-American, September 8.

[R7] Beach Brian (2022) “Water infrastructure and health in US cities.” Regional Science and Urban Economics 94: 103674.

[R8] Beach Brian, Clay Karen, and Saavedra Martin (2022) “The 1918 influenza pandemic and its lessons for COVID-19.” Journal of Economic Literature 60 (1): 41–84.

[R9] Beach Brian, Ferrie Joseph, Saavedra Martin, and Troesken Werner (2016) “Typhoid fever, water quality, and human capital formation.” The Journal of Economic History 76 (1): 41–75.

[R10] Cutler David, Deaton Angus, and Lleras-Muney Adriana (2006) “The determinants of mortality.” Journal of Economic Perspectives 20 (3): 97–120.

[R11] Cutler David M. (2004) Your Money or Your Life: Strong Medicine for America’s Health Care System. Oxford: Oxford University Press.

[R12] Cutler David M., and Miller Grant (2005) “The role of public health improvements in health advances: the twentieth-century United States.” Demography 42 (1): 1–22. 15782893 10.1353/dem.2005.0002

[R13] Deaton Angus (2013) The Great Escape: Health, Wealth, and the Origins of Inequality. Princeton: Princeton University Press.

[R14] Feigenbaum James J., Hoehn-Velasco Lauren, Muller Christopher, and Wrigley-Field Elizabeth (2022) “1918 every year: Racial inequality in infectious disease mortality, 1906−1942.” AEA Papers and Proceedings (112): 199–204.

[R15] Feigenbaum James J., Muller Christopher, and Wrigley-Field Elizabeth (2019) “Regional and Racial Inequality in Infectious Disease Mortality in U.S. Cities, 1900–1948.” Demography 56 (4): 1371–1388. 31197611 10.1007/s13524-019-00789-z PMC7258300

[R16] Ferrie Joseph P., and Troesken Werner (2008) “Water and Chicago’s mortality transition, 1850–1925.” Explorations in Economic History 45 (1): 1–16.

[R17] Fogel Robert W. (2004) The Escape from Hunger and Premature Death, 1700–2100: Europe, America, and the Third World. Cambridge, UK: Cambridge University Press.

[R18] Gibson Campbell, and Jung Kay (2002) “Historical census statistics on population totals by race, 1790 to 1990, and by Hispanic origin, 1790 to 1990, for the United States, regions, divisions, and states.” Working paper.

[R19] Hacker J. David, Dribe Martin, and Helgertz Jonas (2023) “Wealth and Child Mortality in the Nineteenth-Century United States: Evidence from Three Panels of American Couples, 1850–1880.” Working paper. 10.1017/ssh.2023.12 PMC11415258 39310815

[R20] Haines Michael R. (2001) “The Urban Mortality Transition in the United States, 1800–1940.” Annales de Démographie Historique 101(1): 33–64.

[R21] Haines Michael R. (2006) “Vital Statistics,” in Carter Susan B., Gartner Scott Sigmund, Haines Michael R., Olmstead Alan L., Sutch Richard, and Wright Gavin (eds.) Historical Statistics of the United States: Millennial Edition, Vol. 3. Cambridge, UK: Cambridge University Press.

[R22] Hartman Saidiya (2020) “The End of White Supremacy, An American Romance.” Bomb Magazine, June 5.

[R23] Jácome Elisa, Kuziemko Ilyana, and Naidu Suresh (2021) “Mobility for all: Representative intergenerational mobility estimates over the 20th century.” National Bureau of Economic Research.

[R24] Link Bruce G., and Phelan Jo (1995) “Social conditions as fundamental causes of disease.” Journal of Health and Social Behavior 35: 80–94. 7560851

[R25] McKeown Thomas, and Record R. Graham (1962) “Reasons for the decline of mortality in England and Wales during the nineteenth century.” Population Studies 16 (2): 94–122. 11630508

[R26] Moss R. Maurice (1926) “Adult Life Diseases Take Biggest Toll.” Afro-American, April 10.

[R27] Preston Samuel H., and Haines Michael R. (1991) Fatal years: Child Mortality in Late Nineteenth-Century America. Princeton: Princeton University Press.

[R28] Rawlins E. Elliot (1923) “Marriage in Its Relation to Tubercular People.” New York Amsterdam News, July 11.

[R29] Roberts Samuel Kelton Jr. (2009) Infectious Fear: Politics, Disease, and the Health Effects of Segregation. Chapel Hill, NC: University of North Carolina Press.

[R30] Ruggles Steven, Flood Sarah, Goeken Ronald, Grover Josiah, Meyer Erin, Pacas Jose, and Sobek Matthew (2018) IPUMS Ancestry Full Count Data: Version 3.0 [dataset]. Minneapolis, MN: IPUMS.

[R31] Steckel Richard (1988) “The Health and Mortality of Women and Children, 1850–1860.” Journal of Economic History 48 (2): 333–45.

[R32] Stein Melissa (2012) “‘Nature is the Author of Such Restrictions’: Science, Ethnological Medicine, and Jim Crow,” in Cole Stephanie and Ring Natalie J. (eds.) The Folly of Jim Crow: Rethinking the Segregated South. Arlington, TX: Texas A & M University Press.

[R33] Strong Mary (1932) “Friendly Advice to Girls.” Pittsburgh Courier, June 11.

[R34] Szreter Simon (1988) “The importance of social intervention in Britain’s mortality decline c. 1850–1914: a re-interpretation of the role of public health.” Social History of Medicine 1 (1): 1–38.

[R35] Troesken Werner (2004) Water, Race, and Disease. Cambridge, MA: MIT Press.

[R36] Williams A. Wilberforce (1916) “Tuberculosis and Marriage.” Chicago Defender, April 8.

[R37] Zelner Jon, Muller Christopher, and Feigenbaum James J. (2017) “Racial inequality in the annual risk of Tuberculosis infection in the United States, 1910–1933.” Epidemiology & Infection 145 (9): 1797–1840. 28436340 10.1017/S0950268817000802 PMC9203285

