# Supplementary material for: Racial Inequality in the Prime of Life: Infectious Disease Mortality in U.S. Cities, 1906–1933
Source: Soc Sci Hist. Author manuscript; Available in PMC 2024 Dec 13. (PMC11643600; doi:10.1017/ssh.2023.4)
Supplement: Supp material [file NIHMS2021163-supplement-Supp_material.pdf]

A Appendix–For Online Publication

A.1 Additional Tables and Figures

Table A.1: Age Classification Changes, 1921/1922

Classification	Before 1922	1922 and After
Under 1	Under 1	Under 1
1-4	1-4	1-4
5-9	5-9	5-9
10-19	10-19	10-19
20s	20-29	20-29
30-40s	30-49	30-44
50-60s	50-69	45-64
65+/70+	70+	65+

Table A.2: Cause of Death Classification

Classification	Causes Included
Respiratory	Any variant of influenza, pneumonia, or bronchitis
Childhood	Diphtheria, measles, pertussis/whooping cough, scarlet fever, smallpox
Waterborne and Foodborne	Appendicitis, diarrheal diseases, dysentery, poliomyelitis, typhoid and paratyphoid, and "other diseases of the digestive system"
Tuberculosis (TB)	All forms of TB, for example "tuberculosis of the lungs," "tuberculosis of the meninges"
Other Infectious	Erysipelas, exophthalmic goiter, malaria, meningitis, nephritis/Bright's disease, puerperal fever/puerperal septicemia, syphilis, "all other epidemic diseases" and "all other infective and parasitic diseases"

Table A.3: List of Cities Included In Age-Racial-Group Mortality Series

	City	Year Included
1	New Orleans	1906-1933
2	Baltimore	1906-1933
3	Memphis	1906-1933
4	Louisville	1906-1933
5	Kansas City	1906-1933
6	Washington	1906-1933
7	Boston	1908-1918
8	Pittsburgh	1908-1933
9	Saint Louis	1908-1933
10	Chicago	1908-1933
11	Indianapolis	1908-1933
12	New York	1908-1933
13	Philadelphia	1908-1933
14	Cincinnati	1908-1933
15	San Francisco	1909-1933
16	Nashville	1910-1933
17	Atlanta	1910-1933
18	Birmingham	1910-1933
19	Richmond	1910-1933
20	Los Angeles	1913-1933
21	Columbus	1913-1933
22	Oakland	1914-1915
23	Cleveland	1920-1933
24	Norfolk	1920-1933
25	Dallas	1920-1933
26	Detroit	1920-1933
27	Houston	1920-1933
28	Miami	1930-1933
29	Fort Worth	1930-1933
30	Jacksonville	1930-1933
31	San Antonio	1930-1933
32	Chattanooga	1930-1933
33	Newark	1930-1933
34	El Paso	1930-1933

NOTES: El Paso excluded from the analysis due to excessive infectious mortality.

Table A.4: 1900 City Characteristics, By DRA Entry and Whether Age-Racial-Group Mortality Data Exists

	Entry 1900-1909	Entry 1910-1919	Entry 1920-1929	Entry 1930+
	1900 Mean	1900 Mean	1900 Mean	1900 Mean
**Has Age-Racial-Group Mortality**				
Population (1,000s)	48,934.91	8,518.21	15,951.32	5,725.59
% <1	1.91	1.76	2.04	2.16
% <5	9.58	8.71	10.09	10.17
% 5-19	27.63	28.25	29.12	29.19
% 20-44	45.09	44.72	44.85	45.77
% 45-65	14.22	14.79	13.03	12.43
% 65+	3.47	3.53	2.90	2.44
% Female	50.46	51.41	50.39	49.02
% Reproductive-Age Female	27.42	28.35	27.64	26.97
% White	86.47	75.60	80.34	76.29
% In the South	35.71	57.14	60.00	85.71
**No Age-Racial-Group Mortality**				
Population (1,000s)	3,768.60	1,137.49	774.96	376.02
% <1	2.09	2.03	2.12	2.06
% <5	10.19	10.15	10.44	10.57
% 5-19	28.50	29.37	29.79	30.60
% 20-44	42.36	43.37	42.03	40.18
% 45-65	14.73	13.54	13.62	14.43
% 65+	4.23	3.56	4.11	4.22
% Female	50.60	49.09	49.38	50.22
% Reproductive-Age Female	26.20	25.74	25.73	25.56
% White	96.11	85.80	87.10	86.89
% In the South	6.04	27.50	33.79	34.76

NOTES: Shows the 1900 census characteristics for cities in our main sample ('has age-racial-group mortality' data) and cities that are not included in the age-racial-group series ('no age-racial-group mortality' data). Columns show the characteristics by DRA entry timing. For the sample of cities in our main sample, we use the entry year as the year the city began reporting age-racial-group data, instead of the year the city reported any mortality data. SOURCES: Mortality data by age and racial classification from published volumes of the *Vital Statistics of the United States*.

Table A.5: Reduction in Age-Specific Racial Disparity (%)

	Tuberculosis				*Waterborne*				*Flu/Pneumonia*				*Childhood*			
	Ratio		*Difference*		*Ratio*		*Difference*		*Ratio*		*Difference*		*Ratio*		*Difference*	
Age Category	Mean	Max	Mean	Max	Mean	Max	Mean	Max	Mean	Max	Mean	Max	Mean	Max	Mean	Max
Infant	2.8	4.9	5.1	8.4	11.1	24.9	22.8	40.0	30.2	44.3	53.4	70.4	6.1	25.8	11.2	61.0
1-4	13.1	25.0	22.7	45.3	5.2	10.4	8.1	19.8	30.9	43.4	51.8	73.5	3.6	10.8	5.7	18.0
5-9	31.8	48.2	59.3	89.1	4.1	18.4	6.8	29.4	13.1	35.6	25.3	57.2	-2.6	14.6	-2.3	23.1
10-19	55.5	70.5	73.7	85.7	2.8	8.1	3.7	10.1	11.9	18.6	16.0	29.5	-0.2	1.4	-0.3	1.8
20-29	44.5	59.7	69.2	85.1	1.2	3.2	2.0	5.5	13.6	24.2	20.5	39.3	-0.1	0.3	-0.2	0.4
30-49/30-44	29.3	40.2	51.1	67.2	0.8	2.3	1.3	3.7	16.4	26.1	27.4	40.2	-0.0	0.4	-0.0	0.8
50-69/45-64	14.4	26.5	29.0	62.6	0.8	3.3	1.5	6.3	17.1	24.1	31.7	44.4	-0.0	0.6	-0.1	1.3
65+	4.8	17.6	12.4	59.0	-1.3	3.9	0.8	28.6	1.0	11.9	23.0	79.5	-0.1	0.0	-0.1	2.4
All	24.6	31.5	48.7	60.1	3.0	7.0	5.7	13.0	18.1	28.0	35.4	48.9	1.4	6.2	2.6	11.4

NOTES: We calculate the adjusted disparity by setting the nonwhite age-specific mortality rate to the white age-specific mortality rate for each cause, and then recalculating the age-specific infectious disease mortality rate with that adjusted mortality rate. Then the percent of the racial disparity explained by each cause (for each age) is calculated by comparing the observed disparity to the adjusted disparity, where the disparity is the ratio or the difference of nonwhite and white infectious disease mortality for each age. Specific values calculated by taking median across city and average/max across years.
